# Day Surgery in Older Adults: Safety, Effectiveness, and Best Practices for Patient Selection and Perioperative Care—A Narrative Review

**DOI:** 10.3390/geriatrics11030054

**Published:** 2026-04-28

**Authors:** Judit Groman, Zsolt Viktor Göböl, Andrea Virág, Gyula Domján, Klara Gadó

**Affiliations:** 1 Multidisciplinary Day Surgery Clinic, Faculty of Medicine, Semmelweis University, 1082 Budapest, Hungary; groman.judit@semmelweis.hu (J.G.); gobol.zsolt.viktor@semmelweis.hu (Z.V.G.); 2 Doctoral School of Health Sciences, Faculty of Health Sciences, Semmelweis University, 1088 Budapest, Hungary; virag.andrea@semmelweis.hu; 3 Department of Geriatrics and Centre of Nursing Sciences, Semmelweis University, 1085 Budapest, Hungary; domjan.gyula@semmelweis.hu; 4 Department of Clinical Studies, Faculty of Health Sciences, Semmelweis University, 1088 Budapest, Hungary

**Keywords:** older patients, day surgery, geriatric assessment, prehabilitation, nosocomial infections, patient safety, frailty, multidisciplinary care

## Abstract

Background: The growing number of older adults undergoing surgical procedures requires care models that minimise hospital exposure, optimise safety, and support rapid recovery. Day surgery has become an increasingly attractive option for selected older patients, provided their medical, functional and psychosocial needs are carefully assessed. Recent developments in prehabilitation, geriatric-focused perioperative pathways and enhanced post-discharge follow-up have further expanded its potential. This narrative review aims not only to synthesise current evidence, but also to provide a clinically oriented framework for patient selection, perioperative optimisation, and safe implementation of day surgery pathways in older adults. Main findings: Evidence from the past decade indicates that day surgery can be safe and effective for adults aged ≥65 when supported by structured preoperative assessment, targeted optimisation, and clear discharge criteria. Older patients benefit particularly from reduced risks of hospital-acquired complications, including infection, delirium, immobility and functional decline. Prehabilitation programmes focusing on nutrition, strength, balance and medication review are associated with improved postoperative stability and faster return to baseline function. Multidisciplinary teamwork, integrating surgeons, anaesthetists, geriatricians, nurses, physiotherapists, dietitians and caregivers, play a key role in identifying modifiable risks and ensuring continuity of care. Studies also highlight the value of post-discharge telephone follow-up, caregiver engagement and close collaboration with primary care in preventing readmissions. Conclusions: Day surgery is a viable and patient-centred option for many older adults when careful selection and preparation are combined with age-sensitive perioperative care. Most adverse outcomes can be mitigated through systematic prehabilitation, thoughtful anaesthetic planning, early mobilisation and structured follow-up. The evidence suggests that older patients may benefit from reduced hospital stay, less exposure to harm, and faster functional recovery. Implications for practice: The findings support broader integration of geriatric day surgery into routine care pathways, especially within health systems facing capacity constraints. Clinicians should consider implementing standardised geriatric assessment, multidisciplinary optimisation strategies, and robust discharge and follow-up protocols to enhance safety and effectiveness. With appropriate preparation and coordinated teamwork, day surgery can contribute meaningfully to safer, more efficient and more patient-centred surgical care for older adults.

## 1. Introduction

This narrative review provides an overview of the clinical and organisational principles supporting the inclusion of patients aged 65 years and older in day surgery programmes. In this context, the term “geriatric” is used not strictly as a chronological definition but as a clinical concept referring to older adults with age-related vulnerability, including multimorbidity, frailty, and reduced physiological reserve [1,2]. The article summarises key elements of the geriatric perioperative pathway—from surgical indication and patient selection to discharge and early postoperative follow-up. Particular attention is given to comprehensive geriatric assessment, prehabilitation strategies, perioperative risk mitigation, and the prevention of common hospital-related complications such as delirium and infection. The role of multidisciplinary collaboration and coordinated post-discharge care is also discussed. The aim of this review is to synthesise clinically relevant concepts and emerging evidence to support the safe implementation of day surgery pathways for older adults. Despite the growing body of literature on ambulatory surgery, clear, geriatric-specific guidance on patient selection, perioperative optimisation, and safe implementation of day surgery pathways remains fragmented.

This review addresses this clinical gap by synthesising current evidence and practical considerations to support decision-making in older adults undergoing day surgery, with a particular focus on frailty, functional status, and multidisciplinary perioperative care.

## 2. Literature Search and Selection

This article was conducted as a narrative review intended to summarise current concepts and clinical practices related to day surgery in older adults. Relevant literature was identified through targeted searches of major medical databases, including PubMed and Google Scholar.

Search terms included combinations of the following keywords: “day surgery,” “ambulatory surgery,” “older adults,” “geriatric surgery,” “perioperative care,” “prehabilitation,” “comprehensive geriatric assessment,” and “post-discharge care.”

Boolean operators (AND, OR) were used to combine search terms and refine the search strategy.

Reference lists of relevant publications were also screened to identify additional sources.

Priority was given to peer-reviewed articles, systematic reviews, meta-analyses, clinical guidelines, and key observational studies addressing perioperative care pathways, patient selection, geriatric assessment, and outcomes of ambulatory surgery in older populations. Particular attention was paid to studies examining complications, functional outcomes, and healthcare utilisation in geriatric surgical patients.

Although this study was conducted as a narrative review, efforts were made to enhance methodological transparency and reproducibility. The literature search was primarily focused on publications from January 2010 to January 2025 to reflect contemporary perioperative practices. Only articles published in English were included.

Inclusion criteria comprised studies involving adults aged ≥65 years undergoing day or ambulatory surgery, with a focus on perioperative care, patient selection, geriatric assessment, and postoperative outcomes. Clinical guidelines, systematic reviews, meta-analyses, and high-quality observational studies were prioritised.

Exclusion criteria included studies focusing exclusively on paediatric populations, inpatient-only surgical pathways, or publications lacking clear relevance to geriatric perioperative care.

The selection of sources was guided by clinical relevance, methodological quality, and applicability to real-world perioperative practice. Titles and abstracts were initially screened for relevance, followed by full-text review of selected articles where appropriate.

While a formal systematic review methodology was not employed, the approach aimed to ensure a structured and balanced synthesis of the available evidence.

**Figure 1:**
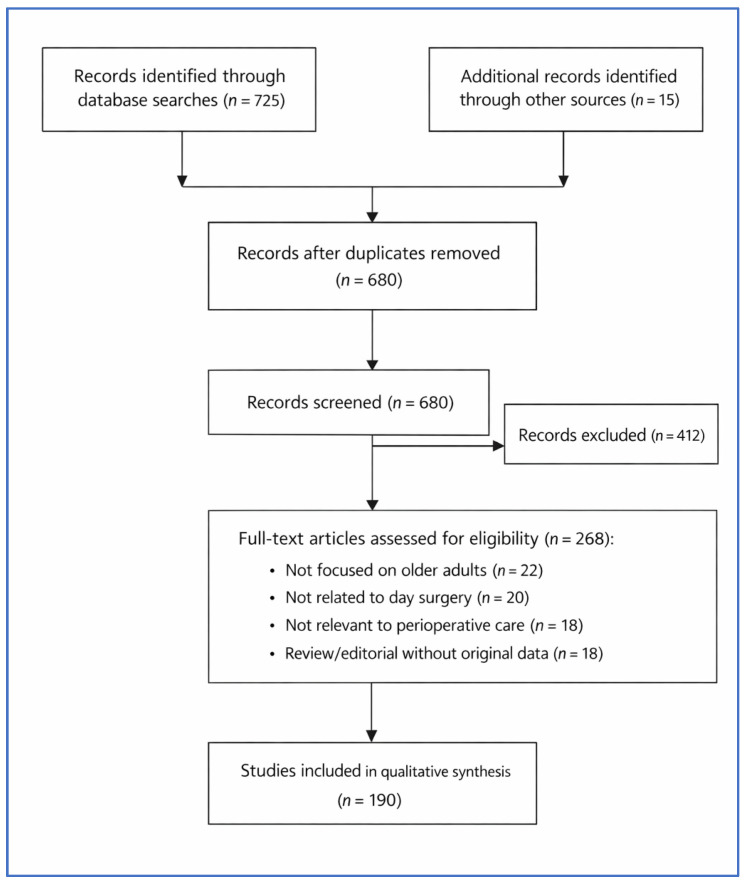
PRISMA-style flow diagram of literature search and study selection process.

This study was conducted as a narrative review and does not follow a formal systematic or scoping review methodology. Therefore, full adherence to PRISMA guidelines was not applicable. However, a PRISMA-style flow diagram was included to enhance transparency in the literature selection process (Figure 1.).

As a narrative review, this approach may be subject to selection bias, which should be considered when interpreting the findings. Potential sources of bias, including selective reporting, publication bias, and heterogeneity in study design and outcome definitions, were considered during interpretation of the findings.

## 3. Background

The reorganisation and optimisation of surgical services in response to this demographic transformation are, therefore, both medical and logistical imperatives. Day surgery has gained increasing prominence as a cost-effective, patient-centred care model that may offer significant advantages in selected older adults [3,4,5,6]. Numerous observational studies and systematic reviews have demonstrated that when applied appropriately, day surgery reduces the incidence of nosocomial complications, enhances recovery trajectories, shortens convalescence, and improves patient satisfaction [5,6].

Historically, older patients have been underrepresented in day surgery cohorts owing to concerns regarding frailty, comorbidity burden, and reduced physiological reserve. However, the emergence of modern perioperative medicine—including enhanced preoperative assessment, structured prehabilitation, minimally invasive surgical techniques, and refined anaesthetic protocols—has markedly improved the safety profile of ambulatory surgery [7]. As a result, older patients, when appropriately selected and optimally prepared, may benefit at least as much as their younger counterparts.

## 4. Terminology and Regulatory Framework of Day Surgery

Day surgery refers to elective surgical procedures in which the patient is admitted and discharged on the same day, with postoperative recovery at home. The goal is to ensure surgical safety while minimising hospital stays, reducing the risk of complications, and utilising healthcare resources efficiently.

According to Hungarian healthcare regulations, day surgery can only be performed if the patient arrives in the morning and is discharged within 24 h of the procedure. Dedicated perioperative monitoring units must meet specific anaesthetic and surgical requirements, and interventions must follow standardised protocols. Patients must receive comprehensive, understandable instructions on home recovery, medication use, recognising complications, and access to follow-up care. Similar regulatory frameworks and organisational principles are applied across many healthcare systems internationally, although the exact administrative requirements and definitions of ambulatory surgery may vary by country [5,6]. Day surgery differs from ambulatory procedures in that it requires more preparation and infrastructure yet does not involve inpatient admission.

### 4.1. Benefits of Day Surgery in Older Patients

Traditional inpatient surgery presents higher risks for older patients, particularly concerning hospital-acquired infections, delirium, and immobility-related complications [3]. In contrast, day surgery, when carefully planned, offers shorter perioperative stress, faster recovery, and lower complication rates [4].

Reduced exposure to hospital environments decreases the risk of infection and delirium [5]; early mobilisation and discharge support physical and mental recovery [6]. Prehabilitation plays a central role in stabilising patients’ condition preoperatively through physical training, nutritional optimisation, medication review, and psychological support [7].

Day surgery may also benefit older adults exposed to polypharmacy due to minimal anaesthesia time and reduced postoperative medication needs [6,7]. Furthermore, it offers logistical and economic advantages, especially in families providing informal care [8,9].

Shorter hospital stays and early mobilisation associated with day surgery also reduce the risk of pressure ulcer development [6]. Prolonged immobilisation is a well-recognised risk factor for pressure injuries in hospitalised older adults; therefore, minimising inpatient time may contribute to improved skin integrity and overall postoperative recovery.

### 4.2. Patient Selection and Risk Stratification

Careful patient selection and structured perioperative risk stratification are central to the safe implementation of day surgery in older adults. Decisions should consider not only the technical feasibility of the surgical procedure but also the patient’s physiological reserve, functional status, cognitive capacity, and available social support [8,9,10].

Chronological age alone should not be considered an exclusion criterion. Instead, a multidimensional evaluation is recommended to determine whether a patient can safely undergo a same-day surgical procedure and recover at home [10].

Comprehensive geriatric assessment tools and screening instruments can support this process. In addition to traditional surgical risk scores such as the ASA classification, geriatric-specific tools may help identify vulnerability and guide perioperative optimisation. Commonly used instruments include the G8 screening tool, the Clinical Frailty Scale (CFS), the Barthel Index for functional status, and cognitive screening tests such as the Mini-Cog, MMSE, or MoCA [11,12] (Table 1).

Medication review using tools such as the STOPP/START criteria may also help reduce perioperative risk [12].

When implemented at the earliest opportunity—ideally during the surgical planning period—comprehensive geriatric assessment facilitates informed decision-making [13,14], enables the timely initiation of prehabilitation, and supports realistic discussions about postoperative expectations [14,15].

This phase encompasses a range of interventions, including medical stabilisation, medication reconciliation, patient education, and strategies aimed at enhancing functional status, such as physiotherapy or nutritional supplementation [9]. In older adults, this preoperative optimisation phase may be as critical as the surgical act itself, particularly in reducing the risk of postoperative complications such as delirium, infection, deconditioning, and functional decline [9].

Ideal candidates for day surgery are typically older adults with preserved functional status, stable chronic conditions, appropriate medication management, and reliable social support. Many ophthalmologic, gynaecologic, and minor general surgical procedures can be performed safely within day surgery pathways in this population [16].

Conversely, day surgery may be inappropriate for patients with severe frailty (Clinical Frailty Scale 6-9), advanced dementia, decompensated cardiac or renal disease, poorly controlled metabolic conditions, or insufficient postoperative support at home. In such cases, inpatient care may provide a safer environment for perioperative monitoring and recovery [16].

### 4.3. Proposed Clinical Algorithm for Patient Selection in Geriatric Day Surgery

Based on the synthesis of current evidence and clinical practice considerations, a structured approach to patient selection in geriatric day surgery is proposed.

The decision-making process should begin with initial frailty screening using a rapid tool such as the Clinical Frailty Scale. Patients identified as frail should undergo comprehensive geriatric assessment, including evaluation of functional status (e.g., Barthel Index), cognitive function (e.g., Mini-Cog), nutritional status (e.g., Mini Nutritional Assessment), and medication review (e.g., STOPP/START criteria).

Following this multidimensional assessment, patients can be stratified into low-, intermediate-, and high-risk categories. Low-risk patients may proceed to day surgery with standard perioperative care. Intermediate-risk patients may benefit from targeted prehabilitation and optimisation prior to surgery. High-risk patients, particularly those with severe frailty or unstable comorbidities, should be considered for inpatient surgical pathways.

Social support and postoperative care capacity should be evaluated at each stage, as inadequate home support may represent a contraindication to day surgery regardless of medical fitness.

This structured approach may support more consistent clinical decision-making and improve patient safety by aligning surgical pathways with individual risk profiles.

The Role of Prehabilitation in Surgical Preparation of Older Patients.

Prehabilitation has emerged as an important strategy to improve surgical outcomes in older adults by enhancing physical, nutritional, and psychological readiness before surgery. Rather than focusing solely on postoperative recovery, prehabilitation aims to strengthen physiological reserve and functional capacity in the weeks preceding the intervention. This approach is particularly relevant in older adults, where frailty, sarcopenia, and multimorbidity increase vulnerability to postoperative complications [13,14].

The primary objective of prehabilitation is to reduce the risk of adverse postoperative events, including delirium, infection, deconditioning, and functional decline, while supporting faster recovery and return to baseline function [15]. Structured multimodal programmes, including evidence from randomized trials and observational cohort studies, have been associated with fewer postoperative complications and improved recovery in frail and pre-frail older surgical patients [17]. Most prehabilitation programmes described in the literature are implemented for approximately two to four weeks prior to surgery, although the optimal duration may vary depending on the patient’s baseline functional status and the type of surgical procedure [13,14,15,17].

Multimodal prehabilitation programmes commonly include several components.

Physical training. Exercise interventions aim to improve strength, balance, and endurance. Activities such as sit-to-stand exercises, gait training, and respiratory exercises can support postoperative mobilisation and reduce pulmonary complications [13].

Nutritional optimisation. Malnutrition and sarcopenia are common among older adults and are associated with impaired wound healing and delayed recovery. Nutritional screening tools such as the Mini Nutritional Assessment can help identify deficits early. Interventions may include protein-rich diets, oral nutritional supplementation, and targeted micronutrient support [14].

Medication review. Polypharmacy and potentially inappropriate medications increase perioperative risk. Medication reconciliation and deprescribing strategies may reduce complications such as delirium, hypotension, or bleeding [18].

Psychological preparation and patient education. Clear communication about the surgical process helps reduce anxiety and improve adherence to perioperative instructions. Involving caregivers in the preparation process can also support postoperative monitoring and recovery [19,20].

Family and caregiver engagement. Older adults often rely on family support during recovery. Involving caregivers early in the preparation process ensures continuity of care, facilitates safe discharge planning, and enhances home monitoring [20].

In some healthcare settings, rehabilitation pathways are organised through multidisciplinary collaboration, including surgeons, anaesthesiologists, physiotherapists, dietitians, pharmacists, nurses, and geriatricians. This team-based approach enables tailored interventions informed by comprehensive geriatric assessments and ensures that the preoperative phase is an integral part of the patient’s recovery trajectory [21].

### 4.4. Indications and Contraindications for Day Surgery in Older Patients

Day surgery is increasingly feasible in older adults, but careful patient selection remains essential. Ideal candidates are typically between 65 and 80 years of age, with preserved cognitive and functional status, stable chronic conditions, appropriate medication management, and reliable social support.

Procedures such as ophthalmologic, gynaecologic, and minor general surgical interventions can often be performed safely in this population. Patients with a history of hospital-related complications, including infections, delirium, or functional decline, may particularly benefit from minimising hospitalisation and avoiding prolonged inpatient stays [3,4,5,6].

However, day surgery may not be appropriate for certain high-risk individuals. Frail patients (Clinical Frailty Scale 6-9), those with advanced dementia, decompensated heart or renal failure, poorly controlled diabetes, or insufficient postoperative support at home may require inpatient management to ensure safe perioperative monitoring and recovery [16].

In addition to clinical factors, environmental and organisational aspects of the surgical pathway can influence the safety of day surgery for older adults. Age-friendly design elements—such as clear signage, adequate lighting, high-contrast visual cues, and well-organised recovery areas—may facilitate safer navigation of the perioperative environment and support smoother recovery.

For patients who do not have an established primary care physician, surgical teams may need to arrange alternative follow-up strategies, such as structured telephone follow-up, outpatient surgical reviews, or coordination with community nursing services to ensure continuity of care after discharge.

### 4.5. Administrative and Logistical Preparations

Preoperative days involve scheduling, obtaining informed consent, patient education, and assessing the home environment [22]. Family involvement is crucial for older adults [21]. Written postoperative instructions should cover pain control, wound care, warning signs, and follow-up [16].

Discharge planning must begin before surgery. Only patients with sufficient home monitoring and caregiver availability should undergo day surgery [23].

### 4.6. The Day of Surgery–Considerations for Older Adults

Older patients have a higher prevalence of cognitive impairment, all of which must be considered during perioperative planning [24]. Older patients typically arrive at the surgical facility early in the morning, allowing sufficient time for careful evaluation and preoperative preparation. Vital signs, medication compliance, hydration status, and cognitive orientation are reassessed before the procedure. In many centres, a preoperative geriatric checklist is used to confirm that all necessary criteria are met before proceeding [25].

Anaesthetic management is tailored to the patient’s functional and medical status. Whenever feasible, minimally invasive techniques such as local, regional, or short-acting general anaesthesia are favoured to minimise cognitive and haemodynamic side effects [26]. The use of short-acting agents reduces the risk of postoperative confusion, prolonged sedation, and delayed discharge. Anaesthesiologists experienced in geriatric care play a crucial role in selecting the safest and most suitable regimen [27].

Throughout the procedure and the immediate recovery period, older adults benefit from continuous and age-aware monitoring. This includes not only standard vital sign observation but also pain assessment, mobility evaluation, and attention to signs of delirium, hypothermia, or orthostatic hypotension. Special care is taken to ensure proper positioning, prevent pressure sores, and maintain thermal comfort, as older patients are more susceptible to environmental stressors [28].

Early postoperative mobilisation is encouraged as soon as the patient is stable, usually within hours after surgery. Mobilisation helps reduce the risk of deep vein thrombosis, pulmonary complications, and delirium, and it facilitates a return to baseline functional status [6,15]. Similarly, oral fluid and light food intake are reintroduced as tolerated, which supports gastrointestinal function and enhances patient comfort.

Discharge is not dictated by the clock but by strict, evidence-based criteria. The patient must be haemodynamically stable, alert and oriented, able to void, mobilise safely, and tolerate oral intake. Pain must be controlled with oral medications, and there must be adequate support available at home, including a responsible adult caregiver. In some centres, a brief functional test—such as sitting, standing, walking, and using the toilet independently—is conducted before clearance [26,28].

Importantly, discharge education is tailored to the cognitive and sensory abilities of older adults, with clear written instructions and verbal reinforcement. Family members or caregivers are involved in this process to ensure understanding and adherence to the treatment plan.

Postoperative follow-up is an integral component of day surgery in older patients’ protocols. Structured telephone calls are conducted within 24–72 h to assess pain control, wound status, mobility, and overall well-being. If concerns arise, patients are referred to their general practitioner or, where available, a home nursing service. In selected cases-—particularly among frail or socially isolated patients—planned in-person follow-up visits may be arranged to prevent complications and reassure both the patient and their family [29].

### 4.7. The Role of Multidisciplinary Teamwork

Safe and effective day surgery in older adults is not solely the result of individual clinical expertise but rather the product of coordinated multidisciplinary teamwork. As older patients often present with complex health profiles—including multiple comorbidities, polypharmacy, cognitive impairment, and varying levels of physical function—their care requires close collaboration among diverse healthcare professionals throughout the perioperative period [30].

At the core of this team are surgeons and anaesthesiologists, whose roles go beyond performing the procedure and administering anaesthesia. Their contribution includes early identification of surgical risks, preoperative counselling, and close coordination with other specialities to tailor the procedure and anaesthetic plan to the patient’s risk profile [30].

Internists and geriatricians play a pivotal role in assessing and optimising chronic conditions such as cardiovascular disease, diabetes, and cognitive decline. Increasing evidence highlights the central role of geriatricians in the perioperative care of older adults, particularly through comprehensive geriatric assessment and multidisciplinary perioperative planning. They guide preoperative medication adjustments, evaluate functional status and frailty, and contribute to risk stratification using both clinical judgement and structured assessment tools. Their involvement is also essential in discharge planning and coordination of postoperative rehabilitation needs [31].

Nurses, especially those trained in perioperative geriatrics or ambulatory care, serve as key coordinators. They monitor vital signs, administer medications, support patient education, and assess post-anaesthesia recovery. In day surgery settings, nurses often take the lead in ensuring that discharge criteria are met and that patients and caregivers are fully informed about postoperative care [32].

Physiotherapists contribute to prehabilitation and early mobilisation strategies, helping to prevent deconditioning, falls, and pulmonary complications. Preoperative assessment by a physiotherapist can inform whether the patient is physically suitable for same-day discharge or if they require further functional support [33].

Dietitians help address malnutrition and sarcopenia—both common in older adults—through targeted nutritional interventions. Preoperative nutritional optimisation has been shown to improve wound healing, immune response, and postoperative outcomes [7].

Pharmacists support safe prescribing by identifying potentially inappropriate medications (PIMs), managing drug interactions, and advising on perioperative adjustments, particularly in patients taking anticoagulants, antihypertensives, or hypoglycaemics [34].

Beyond the clinical staff, administrative personnel also contribute to the success of day surgery by ensuring accurate scheduling, facilitating timely communication between departments, coordinating preoperative assessments, and managing follow-up logistics.

Geriatric day surgery should be guided by interdisciplinary care pathways that support shared decision-making, with patients—and, where appropriate, caregivers—actively involved in the planning process [35]. Decision-making should be informed not only by surgical feasibility but also by patient preferences, life expectancy, functional goals, and the anticipated impact on quality of life [35].

### 4.8. Patient Safety in Geriatric Day Surgery

Patient safety is a central concern in all surgical care. However, it is particularly critical in the context of geriatric day surgery, where the balance between medical efficiency and individualised patient needs must be carefully maintained. Older adults often present with complex health profiles, including multiple comorbidities, polypharmacy, and varying degrees of functional or cognitive impairment. These factors necessitate a comprehensive and structured approach to preoperative risk assessment and perioperative management to ensure optimal outcomes and prevent avoidable complications.

A cornerstone of safe day surgery in older adults is the implementation of structured geriatric assessments. Tools such as the Comprehensive Geriatric Assessment (CGA) can identify frailty, cognitive dysfunction, depression, malnutrition, and functional decline—factors that are often under-recognised yet strongly predictive of postoperative complications, prolonged recovery, and readmission [5,11,21]. This multidimensional evaluation supports more accurate risk stratification and allows for tailored perioperative planning.

Preoperative medication review is another essential component of patient safety. Older adults are more likely to be taking potentially inappropriate medications (PIMs) or to be at risk of drug–drug interactions and adverse drug events. Reviewing and adjusting medications—particularly anticoagulants, sedatives, and anticholinergic agents—can significantly reduce the risk of postoperative delirium, bleeding, hypotension, or falls. Collaboration with clinical pharmacists or geriatricians can further optimise pharmacological safety [36].

Delirium prevention is a priority in the perioperative care of older patients. Delirium is common even after minor procedures and is associated with longer recovery, increased morbidity, and reduced quality of life. Non-pharmacological interventions—such as ensuring proper hydration, pain control, orientation strategies, and minimising unnecessary sensory deprivation—are key measures [37]. Avoidance of high-risk medications and early mobilisation are also effective in reducing incidence.

Strict adherence to evidence-based discharge criteria is crucial in day surgery settings. Patients must be haemodynamically stable, pain-controlled with oral medications, mentally alert, and functionally able to mobilise and manage self-care or have adequate caregiver support. Premature discharge may compromise safety, while overly cautious discharge can reduce the efficiency of ambulatory care.

Finally, effective education of both patients and caregivers enhances safety by promoting vigilance and adherence to postoperative instructions [19,20]. Verbal and written information should be age-appropriate, straightforward, and reinforced at multiple points of contact. Patients and families who are well-informed are more likely to recognise early warning signs of complications—such as infection, bleeding, or confusion—and to seek timely care.

### 4.9. Preventing Nosocomial Infections Through Day Surgery

One of the key advantages of day surgery, particularly in older adults, is the significantly lower risk of healthcare-associated (nosocomial) infections. By minimising inpatient exposure and facilitating early discharge, day surgery substantially reduces the patient’s contact with hospital-acquired pathogens. This is especially important in older adults, who often have reduced physiological reserves and are more susceptible to infections due to age-related immune system decline and multiple comorbidities [38].

Nosocomial infections such as surgical site infections, urinary tract infections—often associated with catheter use—and hospital-acquired pneumonia remain among the most frequent and severe postoperative complications in hospitalised patients. These risks increase in proportion to the length of stay and the number of invasive devices used. In contrast, the day surgery model limits exposure by shortening the time spent in a healthcare environment, typically to a few hours, and by avoiding routine use of catheters, central lines, or extended postoperative monitoring devices [39].

Furthermore, the transition to home-based recovery reduces the likelihood of acquiring multidrug-resistant organisms that are prevalent in inpatient settings. Early mobilization—encouraged as part of standard postoperative protocols in ambulatory surgery—also plays a preventive role by supporting respiratory function, reducing the risk of hypostatic pneumonia, and promoting normal urinary flow, thereby lowering the risk of infection.

Studies have confirmed that infection rates are significantly lower among older patients treated in day surgery settings compared to those undergoing the same procedures during inpatient hospital stays. For example, data from large surgical registries such as the NSQIP (National Surgical Quality Improvement programme) suggest that older adults undergoing ambulatory procedures have up to 50% lower rates of surgical site infections [40].

### 4.10. Post-Discharge Care and the Role of Primary Care

Successful recovery following day surgery in older adults does not end at discharge; it relies heavily on the quality and coordination of post-discharge care. Given that older adults are often discharged on the same day of surgery, frequently within hours of the procedure, it is essential that the transition from hospital to home be seamless, safe, and supported by a reliable care structure [41].

Procedure-specific postoperative management should primarily remain the responsibility of the surgical team. Surgeons and surgical units are best positioned to evaluate procedure-related complications and determine whether additional clinical assessment is required. Timely communication between the surgical team and the patient’s general practitioner (GP) is therefore essential to ensure that primary care providers are informed about the procedure performed, postoperative instructions, medication changes, and potential warning signs [42].

Primary care clinicians play an important complementary role in the recovery process, particularly in managing chronic comorbidities, reviewing medications, and ensuring continuity of care following discharge. This collaboration supports early recognition of health deterioration and facilitates appropriate referral back to the surgical team when necessary [42].

Family or informal caregiver support is another critical factor in home-based recovery [20]. Many older adults require assistance with mobility, medication adherence, wound care, or recognising early signs of complications such as infection, bleeding, or delirium. A caregiver who has been adequately informed during the discharge process is better equipped to monitor the patient and respond appropriately.

In settings where available, home nursing services provide a valuable bridge between hospital and primary care. Community nurses can assess the surgical site, monitor vital signs, manage dressings or catheters if necessary, and provide basic rehabilitative guidance. These visits not only detect early complications but also offer reassurance to patients and caregivers, reducing unnecessary readmissions [29,43].

Early postoperative follow-up by the surgical team is an essential component of safe day surgery pathways. Structured telephone contact, typically conducted within 24–72 h after discharge, allows early identification of complications and timely escalation when necessary, including additional outpatient evaluation. During such calls, healthcare professionals can assess the patient’s condition, review medication adherence, answer questions, and identify warning signs that may require further clinical assessment or in-person evaluation [29,43].

For patients who do not have an established primary care physician, surgical teams may need to organise alternative follow-up strategies, such as scheduled outpatient reviews, structured telephone follow-up, or coordination with community nursing services to ensure continuity of care after discharge.

In addition, clear written instructions, personalised discharge summaries, and access to a dedicated helpline or outpatient surgical contact point can further support recovery and reduce anxiety [29,43]. For older adults, ensuring clear communication is essential, particularly when cognitive impairment, hearing loss, or low health literacy is present. The use of health-literate patient education materials—such as simplified written instructions, visual aids, or structured discharge checklists—may further improve patient understanding and adherence to postoperative recommendations.

## 5. Limitations and Controversies in Geriatric Day Surgery

Despite the growing body of evidence supporting day surgery in older adults, several limitations and areas of controversy should be acknowledged.

A key concern is selection bias. Many studies on day surgery outcomes in older populations include relatively fit and well-selected patients, often excluding those with advanced frailty, cognitive impairment, or significant comorbidity burden. As a result, reported outcomes may overestimate the safety and feasibility of ambulatory surgery in the broader geriatric population.

Heterogeneity in definitions and outcome measures also limits comparability across studies. Variations in how frailty, functional decline, and postoperative complications are assessed may contribute to inconsistent findings and limit generalisability.

Furthermore, frailty and cognitive impairment remain under-recognised in routine surgical practice, despite their strong association with adverse outcomes. This may lead to suboptimal risk stratification and inappropriate patient selection.

There is also ongoing debate regarding the optimal thresholds for selecting older patients for day surgery, particularly in those with borderline functional status or limited social support. While some centres adopt more restrictive criteria, others advocate for broader inclusion supported by enhanced perioperative monitoring and follow-up.

Finally, much of the current evidence is derived from observational studies, and high-quality prospective data specifically focused on geriatric day surgery pathways remain limited. Further research is needed to clarify optimal patient selection criteria and to evaluate the effectiveness of structured perioperative interventions in this population.

These limitations highlight several important directions for future research. There is a clear need for prospective studies focusing specifically on older and frail populations undergoing day surgery, as well as for the development and validation of standardised patient selection algorithms.

Further research should also aim to establish consistent definitions and outcome measures, particularly in relation to frailty, functional recovery, and patient-reported outcomes. In addition, the effectiveness of structured perioperative interventions, including prehabilitation and coordinated post-discharge care models, should be evaluated in well-designed clinical studies.

## 6. Conclusions

Taken together, the available evidence suggests that the safety and effectiveness of day surgery in older adults are not determined by chronological age alone, but rather by a complex interplay of frailty, functional status, comorbidity burden, and the quality of perioperative care.

Across studies, three key elements consistently emerge as central to safe implementation: structured geriatric assessment, targeted prehabilitation, and coordinated multidisciplinary perioperative pathways extending beyond hospital discharge. These components form a practical framework for identifying suitable candidates and optimising outcomes.

Day surgery represents a safe and efficient model of care for many older adults when careful patient selection is combined with comprehensive perioperative optimisation and well-coordinated follow-up. In this context, successful implementation depends on a shift from age-based exclusion towards individualised, risk-based decision-making, while maintaining safety, and may also support broader inclusion of higher-risk patients when appropriate perioperative support is ensured.

From a clinical perspective, early multidimensional assessment—including frailty, functional status, and cognition--together with tailored optimisation strategies should be prioritised. Equally important is the establishment of robust post-discharge care pathways to ensure continuity of care and timely recognition of complications.

Despite these promising findings, the current evidence is largely based on observational studies and heterogeneous populations, which may limit generalisability. Future research should focus on the prospective validation of patient selection algorithms, standardisation of outcome measures, and evaluation of integrated perioperative care models specifically designed for older adults.

In practical terms, the available evidence supports a structured, multidisciplinary approach to geriatric day surgery, in which careful patient selection, targeted prehabilitation, and coordinated perioperative care are central to achieving safe and effective outcomes.

This framework may serve as a practical guide for clinicians aiming to integrate geriatric principles into ambulatory surgical pathways.

## Figures and Tables

**Table 1:** Examples of assessment tools used in geriatric perioperative evaluation.

Domain Assessed	Example Tool	Purpose in Perioperative Assessment
Frailty	Clinical Frailty Scale (CFS)	Rapid clinical assessment of frailty and physiological reserve
Functional status	Barthel Index	Evaluation of independence in activities of daily living
Cognitive function	Mini-Cog, MMSE, MoCA	Screening for cognitive impairment and delirium risk
Nutritional status	Mini Nutritional Assessment (MNA)	Identification of malnutrition and sarcopenia risk
Medication review	STOPP/START criteria	Identification of potentially inappropriate medications and prescribing omissions

## Data Availability

No new data were created or analyzed in this study.
